# Identification and Characterization of a Novel Hemolysis-Related Gene in *Streptococcus* *suis* Serotype 2

**DOI:** 10.1371/journal.pone.0074674

**Published:** 2013-09-17

**Authors:** Jun-xi Zheng, Yue Li, Hui Zhang, Hong-jie Fan, Cheng-ping Lu

**Affiliations:** 1 Key Laboratory of Animal Bacteriology, Ministry of Agriculture, Nanjing Agricultural University, Nanjing, China; 2 Jiangsu Co-innovation Center for Prevention and Control of Important Animal Infectious Diseases and Zoonoses, Yangzhou, China; University of Iowa Carver College of Medicine, United States of America

## Abstract

*Streptococcus* *suis* serotype 2 (SS 2) is an important zoonotic pathogen that has caused two major infectious outbreaks of streptococcal toxic shock syndrome (STSS) in China. A novel gene located in the 89K pathogenicity island (PAI) encoding a putative hemolysin-III-related protein (Hhly3) has been previously characterized. In this study, the SS2 deletion mutant of the exogenous gene *hhly3* was constructed by homologous recombination. This protein was found to exhibit cytolytic activity, and hemolytic activity of the *hhly3* gene knockout mutant (Δ*hhly3*) was significantly lower than that in the wild-type strain ZY05719. In addition, qRT-PCR revealed that Hhly3 played an important role in the expression of the secreted hemolysin SLY, which may be the key reason for the decreased hemolytic activity. Consequently, compared with the WT strain, the infection and pathogenicity of Δ*hhly3* was also decreased, as evidenced by *in vitro* bacterial growth in whole blood and by the *in vivo* zebrafish test, suggesting that *hhly3* is a novel exogenous hemolysis-related gene in SS2 strains.

## Introduction

*Streptococcus* *suis* is a pathogen that is responsible for a variety of life-threatening infections in pigs and humans worldwide. Among the 33 known *S* *. suis* serotypes, *S* *. suis* serotype 2 (SS 2) is one of the most virulent and frequently isolated serotypes [1]. In particular, two major emerging human SS2 outbreaks in China (in 1998 and 2005), which mainly featured streptococcal toxic shock syndrome (STSS), indicated the emergence of new virulent streptococcal variants in China [2]. In our previous study, suppression subtractive hybridization (SSH) was employed to study the virulence factors of SS2 [3]. More than one-third of the hypothetical virulence fragments, including a specific fragment (SSU05_0925), were found to be associated with the candidate pathogenicity island (PAI) termed 89K [4]. This novel gene encoded a protein containing 8 N-terminal transmembrane domains. As the 328-432 aa transmembrane domains were relatively conserved in the integral membrane protein superfamily hemolysin-III (cl03831), this hypothetical protein was represented by the abbreviation Hhly3. Data published to date indicate that the prokaryotic members of the Hly-III family are expressed in many species and that they induce cytolysis of eukaryotic cells via three steps of pore formation [5]. Multiple sequence alignments revealed that Hhly3 showed considerable homology with TrbL/VirB6 plasmid conjugal transfer protein (pfam04610), which was one of the components of type IV secretion system (T4SS) [6]. In this study, an *hhly3* deletion mutant of the Chinese virulent strain ZY05719 was constructed by homologous recombination to determine the role of this putative exogenous gene in hemolysis and other virulence-related functions of SS2.

## Materials and Methods

### Ethics

All animal experimental protocols were approved by approved by the Ethics Committee of Veterinary Institute Jiangsu Academy of Agricultural Sciences (No.SYXK 2010-0005) and performed accordingly. All efforts were made to minimize animals’ suffering.

### Bacterial strains, plasmids, and growth conditions

The bacteria strains and plasmids used in the present study are listed in Table 1 and Table 2. SS2 strains (e.g. ZY05719) were grown in Todd-Hewitt broth (THB, Oxoid) liquid medium and plated on THB agar plates containing 5% (v/v) sheep blood. *E. coli* strains were grown in Luria broth (LB, Oxoid) and plated on LB agar plates. When required, antibiotics were added to the medium at the following concentrations: 100 µg/mL of spectinomycin (Sigma) for the SS2 mutant strain and 100 µg/mL of spectinomycin and 100 µg/mL of ampicillin (Sigma) for *E. coli*.

**Table 1:** Characteristics of the bacterial strains, plasmids, and primers used in this study.

Strain, Plasmid and primer	Relevant characteristics ^a^ or sequence (5′- 3′)^b^		Source or reference
Strains			
ZY05719	Virulent strain isolated from diseased pigs in Sichuan province in 2005		Lab collection
Δ*hhly*3	Isogenic *hhly3* mutant of strain ZY05719		This study
CΔ*hhly*3	Complemented strain of *hhly3*; *Spc* ^R^		This study
*E. coli* DH5α	Host for cloning vector		Invitrogen
*E. coli* BL21 (DE3)	For expressing the recombinant plasmids		Invitrogen
Plasmid			
pSET4s	*S* *. suis* thermosensitive suicide vector; *Spc* ^R^		Takamatsu (2001)
pSET4s: :*hhly3*	A recombinant vector with the background of pSET4s, designed to knockout *hhly3*; *Spc* ^*R*^		This study
pSET2	*E. coli*- *S* *. suis* shuttle vector; *Spc* ^R^		Takamatsu (2001)
pSET2: :*hhly3*	pSET2 containing the intact *hhly3* gene and its upstream promoter; *Spc* ^R^		This study
Primer			
CDS1	GTGAAACCATCAATAGTAAA	Analyze distribution of the *hhly3* gene	This study
CDS2	TCATGGTTTCTTACCGACTT		
RH1	CGGAATTC GTGACCTTTGCAGGAATCTA	Truncated fragment of *hhly3*	This study
RH2	CCCTCGAG ATGGTTTCTTACCGACTTTC		
LA1	AA CTGCAG TTGTAATGGAATCAAC	Upstream border of *hhly3*	This study
LA2	GC GTCGAC AGTTGAAAACTTTAGT		
RA1	CG GGATCC ATTTACACCTCTTTTCGCA	Downstream border of *hhly3*	This study
RA2	CC CCCGGG CTACCAGCAACAGCAATT		
IN-F	GTTGAAATACTAAATGCTGT	Internal region of *hhly3*	This study
IN-R	ACATTAAAAAAATCAATGAC		
OUT-F	CAGCTTGTGCTTCCGATAGTGG	For combined PCR detection	This study
OUT-R	CTTTGGGATTCCTGTGGCTTTC		
hhly3C-F	CGGATCC TTCCGCTGTCCTGTTCATCT	*hhly3* gene and its upstream promoter	This study
hhly3C-R	GCTGCAG CAATCAGAAGCGATTGCGTG		
SLY1	TCATTCAGGTGCTTATGTTGCG	Analyze the expression profiling of *sly* gene	This study
SLY2	GAAGATTGCGAGCATTTCCTGG		

^a^ *Spc* ^*R*^, spectinomycin resistance

^b^ Underlined nucleotides denote enzyme restriction sites.

**Table 2:** Distribution of the *hhly3*-specific DNA fragments.

Strain	Serotype or species	Source^a^	*hhly3*	89K PAI
BA853	SS2	ATCC^a^	-	-
700794	SS2	Canada, ATCC^a^	-	-
P1/7	SS2	Netherlands, ATCC^a^	-	-
T15	SS2	Netherlands, ATCC^b^	-	-
JDZ05802-2	SS2	Jiangxi^a^	+	+
191	SS2	Sichuan^c^	+	+
ZY05721	SS2	Sichuan^a^	+	+
ZY05719	SS2	Sichuan^a^	+	+
SS2-H	SS2	Jiangsu^a^	+	+
HA9801	SS2	Jiangsu^a^	+	+
59A	SS2	Shanghai^a^	+	+
159E	SS2	Shanghai^a^	+	+
97A	SS2	Zhejiang^a^	-	-
JR05730	SS2	Jiangsu^a^	+	+
S50	SS2	Guangdong^a^	-	-
88B	SS2	Guangdong^a^	+	+
227	SS2	Sichuan^c^	+	+
98012	SS2	Jiangsu^c^	+	+
XT	SS2	Hunan^a^	+	+
HA	SS2	Jiangsu^a^	+	+
05464	SS2	Sichuan^a^	+	+
SH28	SS1	Shanghai^a^	-	-
SH13	SS10	Shanghai^b^	-	-
DL15	SS10	Sichuan^b^	-	-
NP4	SS12	N^*a*^	-	-
AH091106	SS11	Anhui^a^	-	-
26S1	SS_1/2_	Canada^a^	-	-
SS9-2083	SS9	Shanghai^b^	-	-
SH06	SS9	N^*a*^	-	-
SH59	SS9	Shanghai^a^	-	-
SH65	SS7	Guangdong^a^	-	-
SH04805	SS7	Shanghai^a^	-	-

^a^ Strains were isolated from diseased pigs.

^b^ Strains were isolated from healthy pigs.

^c^ Strains were isolated from diseased human.

### Distribution of *hhly3* in *S* *. suis* strains and localization of Hhly3

The isoelectric point (pI) and molecular weight (Mw) were predicted using DNAStar software. Primers were designed according to the open reading frame (ORF) to analyze the distribution of *hhly3* in *S* *. suis* strains by PCR (Table 1).

In a previous study, the *shhly3* gene encoding the recombinant truncated protein Hhly3 was amplified using the primers RH1/RH2 (Table 1). The recombinant plasmid pET32a(+)::*shhly3* was transformed into *E. coli* BL21 (DE3) for the expression of the recombinant protein rsHhly3. In addition, the immunogenicity of HisTrap-purified rsHhly3 was analyzed, and anti-rsHhly3 rabbit polyclonal antibody was prepared. In the previous study, Hhly3 was absent in the culture supernatant as well as in the cell wall of the wild-type (WT) strain ZY05719. In this study, the membrane-associated proteins of ZY05719 and its mutants were prepared according to the Triton X-114 extraction protocol [7]. The membrane-associated proteins were detected by Western blot analysis using rabbit polyclonal antibodies to rsHhly3 to determine the localization of Hhly3.

### Deletion and functional complementation of *hhly3*

**Figure 1:**
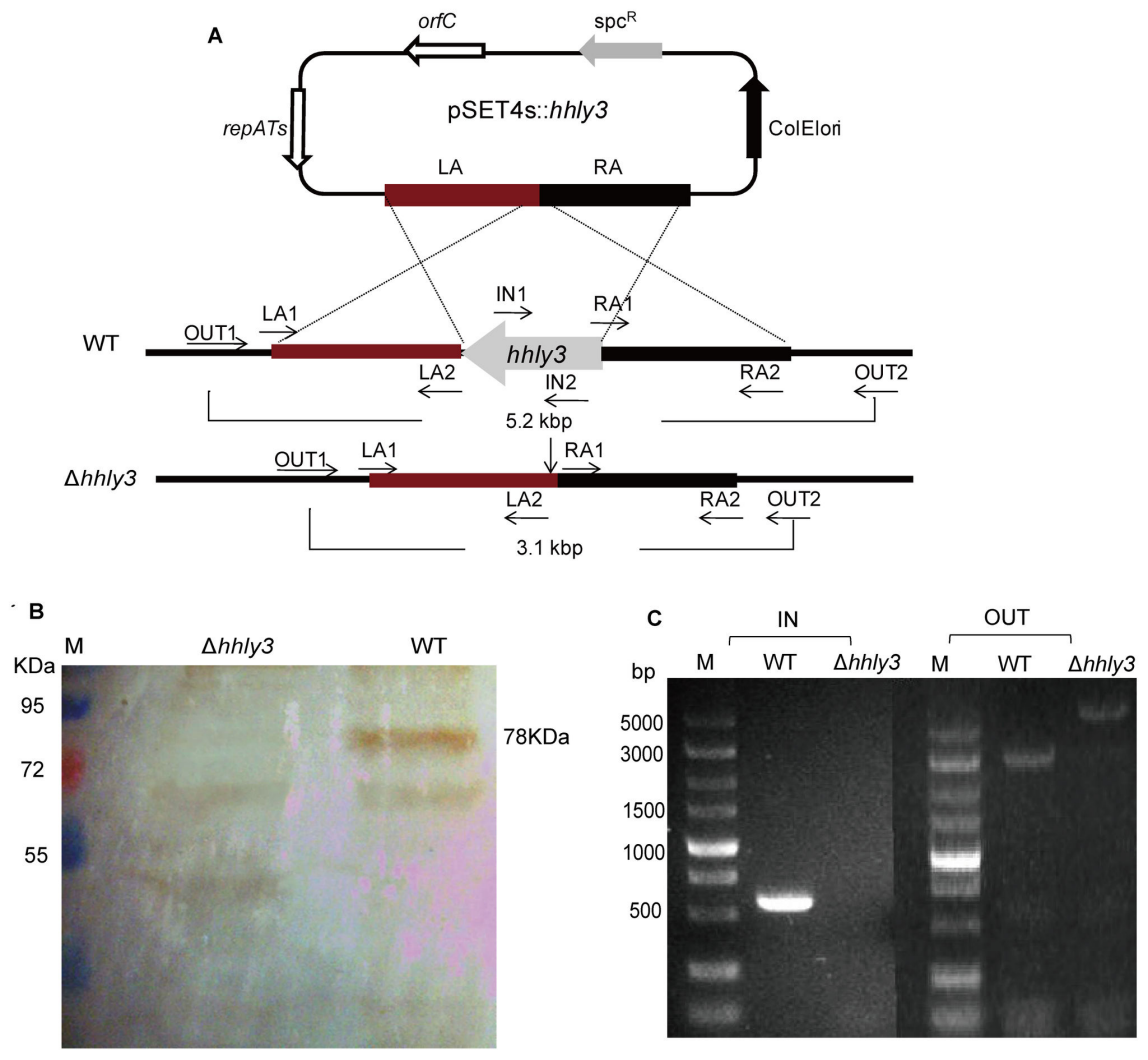
Construction and confirmation of the ZY05719Δ*hhly3* mutant strain. (A) Construction strategy of *hhly3* gene knockout mutant by allelic replacement. (B) Localization analysis of Hhly3. Western blot analysis of membrane-associated proteins with anti-rHhly3 rabbit polyclonal antibodies. (C) PCR confirmation of knockout mutant strain Δ*hhly3*. The specific primer pair IN1/IN2 was used to detect the presence of the *hhly3* gene in the SS2 genome. The flanking primer pair OUT1/OUT2 located outside LA and RA was designed to confirm double-crossover recombination in the mutant by combined PCR detection.

DNA fragments were PCR amplified from the chromosomal DNA of ZY05719 using two pairs of Hhly3-specific primers, LA1/LA2 and RA1/RA2 (Table 1). The fragments were directionally cloned into the temperature-sensitive *S* *. suis* -*E. coli* shuttle vector pSET4s [8] to generate the *hhly3* knockout vector pSET4s::*hhly3*. Procedures for the selection of mutants by allelic exchange via double crossover have been described previously [9]. ZY05719 containing the pSET4s::*hhly3* vector was grown at 28°C in the presence of spectinomycin before screening for the clones both vector-loss and exchanged the wild type allele *hhly3* via a double cross-over homologous recombination at 37°C. The resulting Δ*hhly3* mutant strain was verified by PCR amplification using the primer pairs IN1/IN2 and OUT1/OUT2 (Figure 1A).

For complementation assays, a DNA fragment containing the entire *hhly3* gene and promoter sequence was cloned into the *E. coli*- *S* *. suis* shuttle vector pSET2 [10] to generate the *hhly3*-complementing plasmid pSET2::*hhly3*. The functional complement strain Δ*hhly3* (CΔ*hhly3*) carrying this plasmid was obtained by selective growth in the presence of spectinomycin.

### Hemolysin activity assay

SS2 strains were cultured on sheep blood agar plates incubated in a candle jar to create anoxic conditions. Hemolysin activity was tested as previously described [11]. Briefly, sterile culture supernatants or cell product following ultrasonication were prepared from bacterial cultures and centrifuged to separate the culture supernatant fluids and cell pellets. The sonicated cell pellets were suspended in cold phosphate-buffered saline (PBS; pH 7.0) to obtain bacterial protein. Serial twofold dilutions (150 µL) of the test samples were prepared in 96-well microplates. Approximately 150 µL of 1% sheep erythrocyte suspension was added to THB broth. After incubation for 2 h at 37°C, the microplates were centrifuged at 5,000*g* for 10 min, and 150 µL of the supernatant was transferred to a new plate for spectrophotometric measurements at 540 nm. Hemolysin units (HU) were defined as the reciprocal of the highest dilution of supernatant inducing at least 50% lysis of erythrocytes. The hemolytic activities of culture supernatant fluids during various growth phases were also observed.

### SLY RNA manipulation and qRT-PCR

Total RNA was extracted from the bacterial culture at various growth phases by using the TRIZOL reagent [12]. cDNAs were created by reverse transcription with PrimeScript RT-PCR kit (Takara). The specific primers SLY1/SLY2 of the hemolysin gene *sly* are shown in Table 1. The relative change in the gene expression ratios of the selected genes was normalized to the expression of a single housekeeping gene (16S rRNA gene). Subsequently, the SYBR Premix Ex *Taq* kit (Takara Bio) was used for semi-quantitative RT-PCR assay, and the mRNA levels was analyzed by the comparative cycle threshold method (2 ^–ΔΔCT^ method) [13].

### Survival in the presence of swine whole blood

Blood samples were collected from healthy pigs by venous puncture. Susceptibility assays were performed as previously described [14]. WT, Δ*hhly3*, and CΔ*hhly3* were cultured to OD_600_ = 0.4–0.6, and the cells were pelleted and suspended in THB medium to obtain an OD_600_ of 0.1. The whole blood, pig serum, and SS2 cell mixture was incubated for 3 h at 37°C with occasional gentle shaking. Infected whole blood cultures were harvested at 0 and 3 h to determine bacterial growth using the plate count method. The first time point (0 h) was considered as the 100% viability control.

### Animal infections

The use of zebrafish as an infection model for *S* *. suis* has been accepted by the World Organization for Animal Health (OIE) [15]. Zebrafish were assigned to six groups, with 15 zebrafish per group. As a matter of fact, the median lethal dose (LD50) should be ascertained to determine the differences in virulence between the WT and mutant strains. The SS2 strain was cultured to the logarithmic growth phase, then adjusted to 5 × 10^9^ CFU/mL and serially diluted tenfold until the lowest concentration of 5 × 10^4^ CFU/mL was reached. These three SS2 strains were injected intraperitoneally at a concentration of 0.01 mL into the zebrafish. The mortality of the zebrafish was recorded, and the LD50 value was calculated according to Karber [16].

### Statistical analysis

All assays were performed in triplicate. Statistical analyses were carried out using the GraphPad Software package. Where appropriate, the data were analyzed using Student’s *t* test. The non-parametric Mann-Whitney *U* test was used for the analysis of the animal infection study. A *P* value of <0.05 was considered statistically significant and *P* < 0.01 was considered highly statistically significant.

## Results

### Distribution of *hhly3* in *S* *. suis* strains and localization of Hhly3

The *hhly3* gene was located at 896463–898574 bp on the complementary strand of the ZY05719 genome, and it encoded a hypothetical protein with a predicted molecular mass of 78 kDa and a pI of 9.82. PCR analysis of *hhly3* distribution revealed that this gene was present in most Chinese SS2 isolates, especially those isolated from the regions surrounding the two large outbreak areas, but the gene was not present in the avirulent strain T15 and in other European virulent isolates such as P1/7 (Table 2). This result was also consistent with the detection result of 89K PAI, suggesting that the *hhly3* gene is an exogenous gene of SS2.

Moreover, Western blot analysis of Hhly3 showed that this protein was localized in the membrane of ZY05719 (Figure 1B). As it was absent both in the culture supernatant as well as in the cell wall and its multiple transmembrane domains, we inferred that it may be a membrane channel protein.

### Construction and characterization of an *hhly3*-defective mutant

An isogenic *hhly3* mutant of the WT strain ZY05719 was constructed through homologous recombination. The double-crossover event was confirmed by multiple PCR (Figure 1C) and direct DNA sequencing (data not shown), verifying that an isogenic knockout mutant of *hhly3* (namely Δ*hhly3*) was successfully constructed without alteration of the remaining sequences.

### Deletion of *hhly3* decreases the hemolytic activity of *S* *. suis*

**Figure 2:**
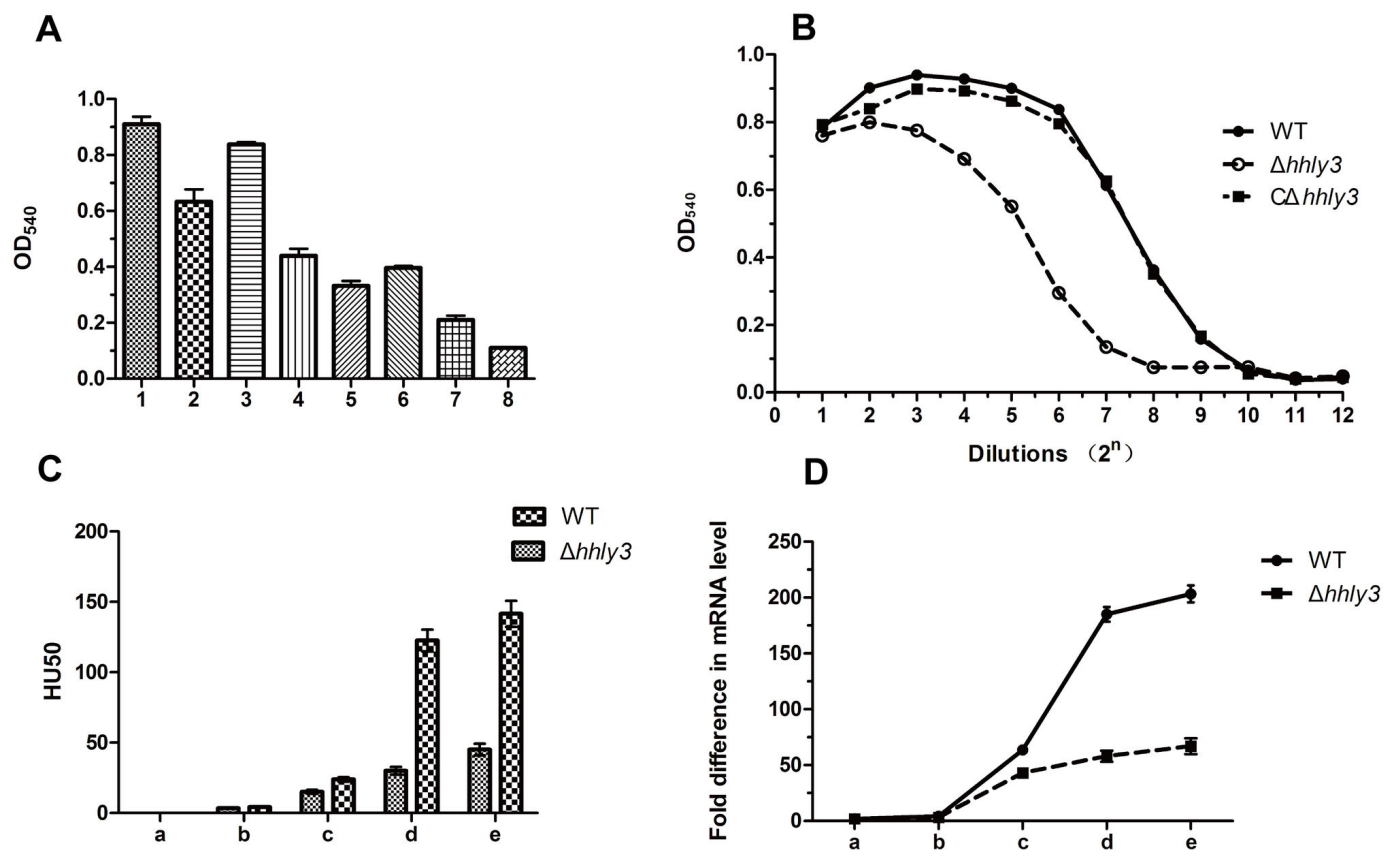
Hemolytic activity analysis. (A) Hemolytic activities of different strains. 1–3, Supernatants of WT, Δ*hhly3*, and CΔ*hhly3* strains, respectively. 4–8, Pellets of WT, Δ*hhly3*, CΔ*hhly3*, BL21 (pSET2::*hhly3*), and BL21 (pSET2) strains, respectively. (B) Titration of hemolytic activities of culture supernatants of different strains in the stationary phase. (C) Hemolytic unit analysis of the supernatants of the SS2 strain at different phases of growth. a, early logarithmic phase (OD_600_ < 0.3). b, mid-logarithmic phase (OD_600_ = 0.4–0.6). c, late logarithmic phase (OD_600_ = 0.6–0.8). d, late logarithmic phase (OD_600_ = 1.0–1.2). e, stationary phase (OD_600_ > 1.2). (D) Expression profiles of *sly* at different stages of growth. a, early logarithmic phase (OD_600_ < 0.3). b, mid-logarithmic phase (OD_600_ = 0.4–0.6). c, late logarithmic phase (OD_600_ = 0.6–0.8). d, late logarithmic phase (OD_600_ = 1.0–1.2). e, stationary phase (OD_600_ > 1.2).

In order to study the hemolytic activity of Hhly3, the *hhly3*-complementing plasmid pSET2::*hhly3* was transformed into BL21 (DE3), the commercially available competent *E. coli* strain. The hemolytic activity of the bacterial protein harvested by ultrasonication was measured. The bacterial protein of pSET2::*hhly3* BL21 had significantly higher (*P* < 0.01) hemolytic activity than BL21 (pSET2; Figure 2A), suggesting that Hhly3 is a hemolytic protein.

In addition, we compared the hemolytic activities of the SS2 mutant strains. Different SS2 strains were inoculated on sheep blood agar plates and incubated in anoxic conditions, and the α-hemolytic zones created by the Δ*hhly3* strain were slightly smaller than those created by the WT strain. Consequently, the hemolytic activity of the supernatant and protein fraction of the Δ*hhly3* mutant was significantly lower (*P* < 0.01) than that in the *hhly3* strain, and this activity was subsequently restored in CΔ*hhly3* (Figure 2A). In general, the hemolysis mainly occurred in the supernatant in the SS2 strain as the hemolytic activities of bacterial proteins were much poorer than those of the culture supernatant (Figure 2A). Furthermore, the effects of *hhly3* inactivation on the hemolytic activities of bacterial proteins were also lower than in the supernatants (Figure 2A).

Considering differences in the hemolytic activities during various growth phases, the late logarithmic phase to stationary phase was considered suitable for determining the hemolytic activity. In the stationary phase (OD_600_ = 1.0–1.3), the highest dilution of culture supernatants that induced at least 50% lysis of erythrocytes for the ZY05719, Δ*hhly3*, and CΔ*hhly3* strains were 1:128, 1:32, and 1:128, respectively (Figure 2B). In terms of cell growth, the difference between the Δ*hhly3* mutant and the WT strain increased in significance till the stationary phase was reached (Figure 2C).

### Expression profiling of the sly gene

The expression profile of *sly* encoding the known secreted hemolysin was analyzed in SS2 strains *in vitro* by qRT-PCR. Equal amounts of total RNA were reverse-transcribed from different growth phases. The expression of *sly* exhibited the same variation as hemolytic activity of culture supernatants (Figure 2d). Compared with the WT strain, the mRNA levels of *sly* in the late logarithmic phase was significantly decreased by 76% in the Δ*hhly3* mutant (*P* < 0.05).

### Decreased susceptibility in whole blood

**Figure 3:**
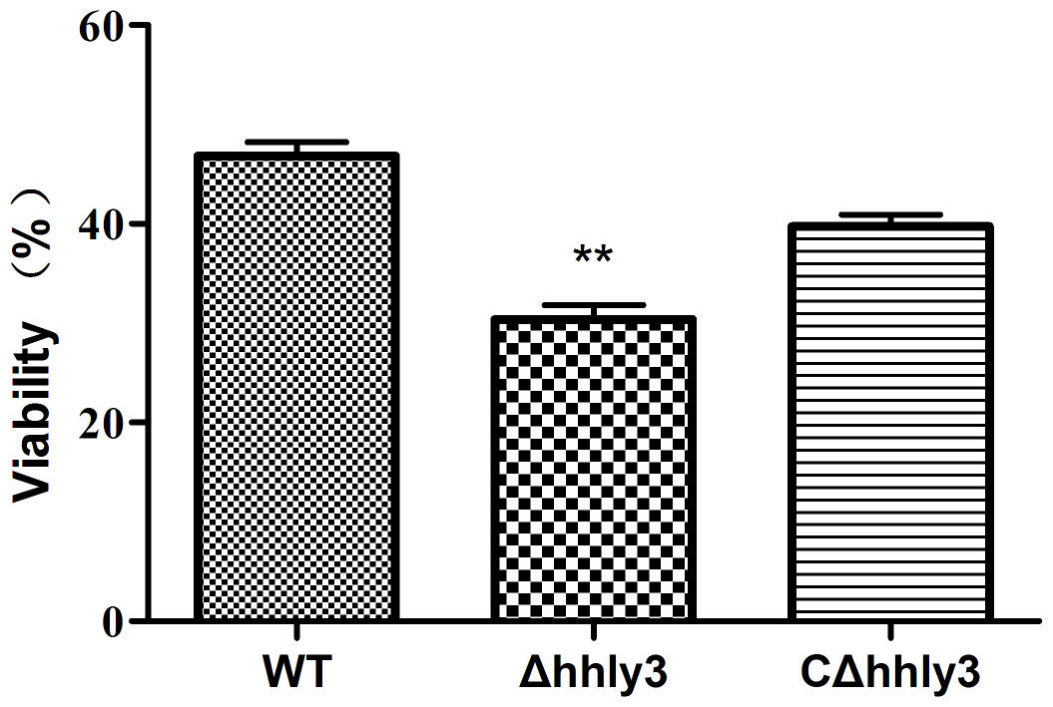
Survival of the WT, Δ*hhly3*, and CΔ*hhly3* strains in pig whole blood. The percentage survival rate of Δ*hhly3* was significantly lower than that in the WT strain and the complement strain CΔ*hhly3* (** *P* < 0.01).

As shown in Figure 3, Δ*hhly3* had a higher death rate in the whole pig blood *in vitro* than WT strain and the complementation strain CΔ*hhly3* (*P* < 0.01). After 3 h of incubation, the survival rates of ZY05719, Δ*hhly3*, and CΔ*hhly3* were 30.4%, 46.8%, 39.7%, respectively, indicating that the resistance of the pathogen to phagocytosis and killing in whole blood decreased by *hhly3* inactivation.

### Virulence attenuation by *hhly3* inactivation

The effect of the *hhly3* gene in virulence was evaluated in the zebrafish model by intraperitoneal administration of various doses of ZY05719, Δ*hhly3*, and CΔ*hhly3*. The mortality of zebrafish was observed within 7 days after the challenge. As shown in Table 3, the LD50 was 2.39 × 10^5^ 1.30 × 10^6^, and 4.23 × 10^5^ CFU/zebrafish for the WT, Δ*hhly3*, and CΔ*hhly3* strains, respectively (*P* < 0.05).

**Table 3:** Calculation of LD_50_ for the ZY05719 strain, mutants, and complemented strain in zebrafish.

Challenge dose (CFU)	Zebrafish numbers	Death numbers ^a^		
		ZY05719	Δ*hhly3*	CΔ*hhly3*
5×10^7^	15	15.0 ± 0.0	15.0 ± 0.0	15.0 ± 0.0
5×10^6^	15	14.0 ± 1.0	10.7 ±1.2	13.3 ± 0.6
5×10^5^	15	9.3 ± 1.2	4.7 ±1.2	8.7 ± 0.6
5×10^4^	15	3.3 ± 0.6	1.0 ± 1.0	1.7 ±0.6
5×10^3^	15	0.7 ± 0.6	0.0 ± 0.0	0.0 ± 0.0
5×10^2^	15	0.0 ± 0.0	0.0 ± 0.0	0.0 ± 0.0
Value of LD_50_ (×10^5^)		2.39 ± 0.21	12.97 ± 1.10^b^	4.23 ± 0.71^b^

^a^ Values are means ± Standard deviations (three samples per group)

^b^ Significantly different (*P* < 0.05) from value for WT strain group as calculated by the non-parametric Mann-Whitney *U* test

## Discussion

Hemolytic activity is a phenotypic marker used to distinguish between highly virulent and avirulent isolates. Thus far, bacterial hemolysin can be categorized into many types, including the cholesterol-binding cytolysin family and repeats in toxin (RTX) family. This study confirmed that Hhly3 possesses hemolytic activity. The hemolytic activity of the Δ*hhly3* mutant was significantly lower than that of the WT strain; however, it did not completely disappear.

This study indicated that SS2 hemolysis was largely dependent on the cytolysis activity of secretory proteins, especially the dominant hemolysin SLY. The characteristics of SLY are clear: this secreted protein had higher expression and secretion levels as well as greater stability in the late logarithmic phase [17,18]. It is noteworthy that as an exotoxin, SLY should be exposed in time after the expression reducing the risk of toxicity to donor bacteria. Hhly3, which has been verified to be a hemolytic membrane channel protein, was considerable to have homology with a component in T4SS for protein secretion [19]. In this study, the low expression of SLY achieved by *hhly3* inactivation was in compliance with the negative regulation to block the secretion of SLY. Therefore, we have reason to speculate that *hhly3* inactivation may block of the secretory pathways of SLY, causing imbalance of expression and secretion. Therefore, the expression level of SLY was reduced to ensure the survival of bacteria, and the hemolytic activity of SS2 was greatly decreased. In addition, we found that the European virulent isolate P1/7, which lacked 89K PAI, showed considerably higher degree of hemolysis while carrying the pSET2::*hhly3* plasmid, indicating that Hhly3 was one of the important factors of cytolysis.

Furthermore, SLY-positive strains seem to additionally benefit from the toxic effects of this hemolysin for polymorphonuclear neutrophil (PMNs) and could better survive in blood [20]. The susceptibility of Δ*hhly3* in pig blood decreased mainly due to the lower expression of SLY, which might account partially for the virulence loss in Δ*hhly3* in zebrafish infection. However, Hhly3 might be also related to the secretion of some extracellular proteins relevant to colonization and immune escape, although additional studies are require to confirm this assumption.

89K PAI is a novel mosaic PAI candidate that is prevalent and unique in Chinese SS2 virulent strains. Since the discovery of this PAI, multiple virulence factors have been characterized [21,22]. This study showed that the putative exogenous gene, *hhly3*, was in 89K PAI and expressed successfully, strongly suggesting that this PAI is responsible for the great variation in the virulence of strain SS2, resulting in high casualties during the two outbreaks of STSS. During the infection of SS2, the WT strain carrying the *hhly3* gene was more significant in infection. More importantly, this hemolytic protein played an important role in expression of the major hemolysis factor SLY, causing considerably greater damage to the host cell.
